# Typification of eight current and seven related names and a new section in the genus *Bromus* (Bromeae, Pooideae, Poaceae)

**DOI:** 10.3897/phytokeys.121.30254

**Published:** 2019-05-02

**Authors:** Félix Llamas, Carmen Acedo

**Affiliations:** 1 Research Group Taxonomy and Biodiversity Conservation TaCoBi, Department of Biodiversity and Environment Management, University of León, E-2407, León, Spain University of León León Spain

**Keywords:** Africa, *
Bromus
*, Europe, nomenclature, new name, typification

## Abstract

During our nomenclatural revision of the genus *Bromus* L. (Poaceae) for Flora Iberica, we found that several names were still untypified: nine names in current use or their basionyms and five synonyms. Typifications are still needed since stabilising the names will facilitate their use. We propose lectotypes for *Bromusalopecuros* Poir., *B.contortus* Desf. (and the superfluous *B.alopecuroides* Poir.), *B.benekenii* (Lange) Trimen, B.intermediusGuss.subsp.divaricatus Bonnier & Layens, *B.molliformis* J.Lloyd ex Billot, *B.lepidus* Holmb., *B.lepidus f. lasiolepis* Holmb., Bromussubg.Stenobromus (Griseb.) Hack. and Bromussect.Stenobromus Griseb. Neotypes for *B.erectus* Huds. and *B.ramosus* Huds. and an epitype for B.intermediussubsp.divaricatus Bonnier & Layens are proposed. In addition, we identify an isoneotype for *B.erectus* and isolectotypes for *B.lepidus* and B.lepidusf.lasiolepis. The area inhabited by the typified taxa includes both Africa and Europe. All the selected types are in agreement with the current use of the names and, thus, our selections contribute to stabilising the nomenclature of the genus *Bromus*. A discussion is provided to justify the selections. In addition, we typified two supraspecific names B.subg.Stenobromus and B.sect.Stenobromus. Finally, a new section, B.sect.Penicillius Llamas & Acedo, is described.

## Introduction

The genus *Bromus* L. (Poaceae) includes about 200 species distributed worldwide, with the greatest diversity and most complex taxonomy in south-eastern Europe and western Asia (Acedo and Llamas 2001). The taxonomy and nomenclature of this genus is difficult and the appropriate ranks of various supraspecific, specific and infraspecific taxa still remain uncertain and contested. In addition, sometimes its sections are raised to genera: *Anisantha* K.Koch, *Boissiera* Hochst. ex Steud., *Bromopsis* (Dumort.) Fourr., *Bromus* L., *Ceratochloa* P.Beauv., *Nevskiella* V.I.Krecz. & Vved. etc. Some authors raise the sections to subgenera (e.g. Stebbins 1981, Acedo and Llamas 1999).

In step with research carried out by various authors in the last decade on the taxonomy and nomenclature of the genus *Bromus* L., we here present a nomenclatural paper concerning the 8 names of well-known and floristically or coenologically important taxa belonging to *Bromus* that remain untypified at present and others related with them.

Besides the basic interest of the typification of the untypified names to stabilise nomenclature, it is valuable for any Flora to include type information and references to the exact places where the designation of types were published. All typifications in our work affect native and naturalised taxa occurring in the Iberian Peninsula, most of them also being present in other European countries and North Africa or have been established as aliens in many territories around the world. In any case, most of these names are applied widely due to their current distribution. We are applying *Bromus* sensu lato circumscription, since there is not sufficient data to split it into different genera.

## Materials and methods

This study is based on analysis of relevant literature (every protologue and location indications included) and search for specimens or images of the following herbaria to identify original material: B, BM, BRI, C, G, FI, H, GOET, L, LD, LE, LEB, LINN, K, MPU, P, PH, PI, S, UPS and W (acronyms according to Thiers 2018+). Finally, by studying digital images or specimens, we designate the most suitable type in each case. All our decisions on typifications follow the rules and recommendations of the *International Code of Nomenclature for algae, fungi and plants* (ICN; Turland et al. 2018).

The references are consulted in the Biblioteca Digital del Real Jardín Botánico de Madrid (2018) at http://bibdigital. rjb. csic.es/ing/index.php, BHL (2017), Biodiversity Heritage Library at https://www.biodiversitylibrary.org/, Botanicus Digital Peter H. Raven Library Missouri Botanical Garden at http://www.botanicus.org/ and Gallica https://gallica.bnf.fr. All available images of specimens can be examined via JSTOR Global Plants (2000–2018) https://plants.jstor.org/ and many on the servers of several of cited herbaria.

Currently accepted names are listed in alphabetical order, including their synonyms in each entry. Accepted names are in italic-bold, while junior synonyms are in italic-non-bold. Specimens seen are marked “!”, images of specimens seen as “image!”.

## Results and discussion

### Bromus alopecuros Poir., Voy. Barbarie. 2: 100–101 (1789)

Taxon classificationPlantaePoalesPoaceae

**Figure 1.**
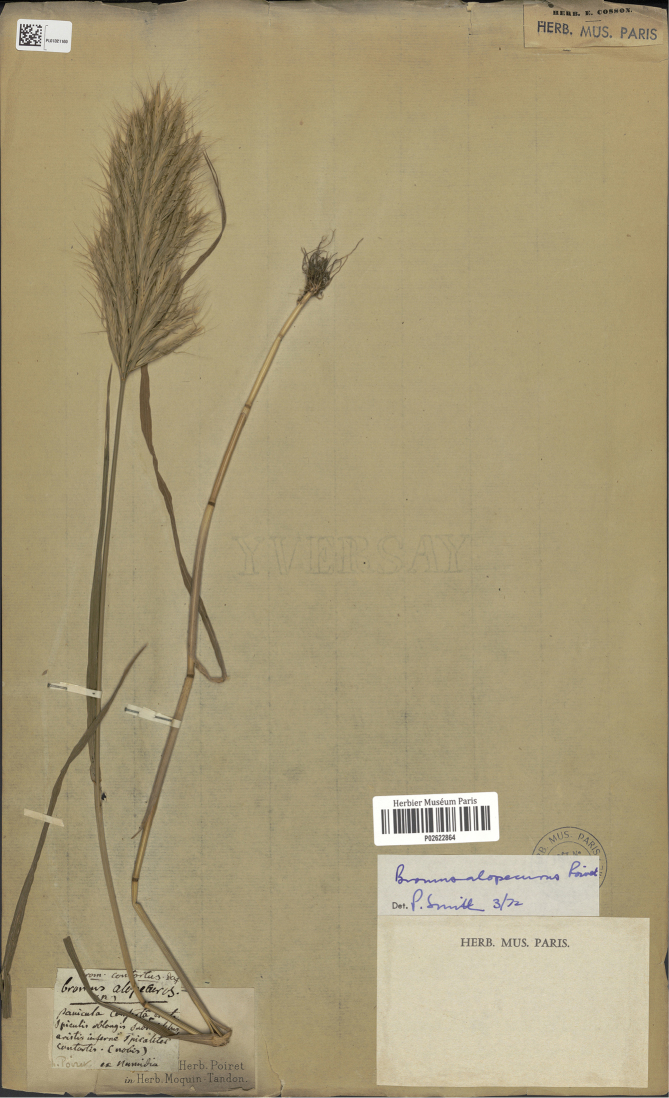
Lectotype of *Bromusalopecuros*: It is a complete specimen preserved at the MNHN Collection Vascular plants Specimen with barcode P02622864. (Available at http://coldb.mnhn.fr/catalognumber/mnhn/p/p02622864).

**Figure 2.**
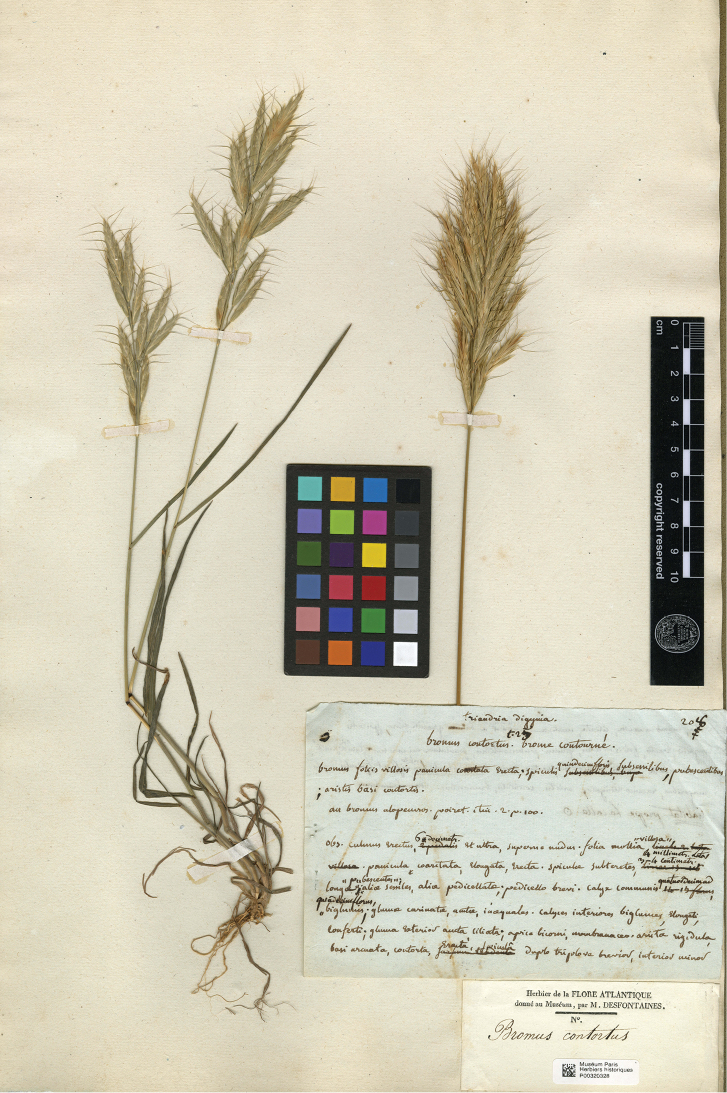
Lectotype of *Bromuscontortus* Desf. is the specimen on the right having subsessile spikelets conserved in the MNHN Collection Vascular plants with barcode P00320328 (image available at: https://science.mnhn.fr/institution/mnhn/collection/p/item/p00320328?listIndex=1&listCount=4).

Bromus alopecuros Poir., Voy. Barbarie. 2: 100–101 (1789). Type Protologue: “Cette espèce croît dans les prairies aux environs de la Calle ”. Type: [Algeria] Numidia (lectotype, designated here: P [P02622864 image!]). (Figure 1)
Bromus contortus Desf., Fl. Atlant. 1: 95, tab. 25 (1798). Type Protologue: “Habitat prope La Calle”. Type: [Algeria] La Calle (lectotype, designated here: P [P00320328 image!]). (Figure 2)
Bromus alopecuroides Poir. in Lamarck, Encycl., Suppl. 1: 703 (1810), nom. illeg. superfl. for *Bromuscontortus* Desf. Type Protologue: [Algeria] “Barbarie, dans les prés, aux environs de Lacalle”.

#### Remarks.

Currently, the species *Bromusalopecuros* Poir. has at least two synonyms: *B.alopecuroides* Poir. and *B.contortus* Desf.

In describing *Bromusalopecuros*, Poiret (1789: 100) stated in the protologue diagnosis “Panicula conferta erecta, spiculis oblongis subsessilibus, aristis inferne spiraliter contortis.” followed by a description in French and the locotypic indication indicating this species grows around La Calle [now El Kala, El Tarf province, Algeria].

During our search for original material in the herbaria conserving the plants of Poiret (FI, H, P and UPS), we found only one specimen collected by him. There is a sheet in P (P02622864) from Numidia (Algeria “Numidia” included in the full title of his publication), registered as original material, bearing a single plant annotated as *B.alopecuros* with five labels transcribed below; one of them handwritten by Poiret:

Label 1: [printed]. Herb. Poiret in Herb. Moquin-Tandom.

Label 2: [handwritten by Poiret] “brom. contortus Desf. *Bromusalopecuros* (n) Panicula conferta erecta spiculis oblongis subsessilibus, aristis inferne spiraliter contortis. (nobis) h. Poiret ex Numidia.

Label 3: [printed]: barcode Herbier museum Paris P 02622864

Label 4: [handwritten] *Bromusalopecuros* Poiret, Det. P. Smith 3/72

Label 5: [printed]. Herb. Mus. Paris

As this specimen bears a label handwritten by Poiret with his description of *B.alopecuros* and the word “nobis” [our], indicating the author is describing a new species, it seems sure that it is original material and it is suitable to be described as a lectotype. In addition, there is no other specimen matching with *Bromusalopecuros* Poir. in FI (Chiara Nepi pers. comm. 2018); nor in H (Raino Lampinen pers. comm. 1994; Henry Väre pers. comm. 2018) nor in UPS (Dr. Mats Hjertson pers. comm. 2018).

When Desfontaines (1798: 95), describes *Bromuscontortus*, his description closely matches that of Poiret (1789). In his diagnosis, Desfontaines adds that the spikelets are “quindecimfloribus, …pubescentibus”. At the end, he has doubts about his plant and transcribes a reference to the description of Poiret “An Bromus alopecuros? Poiret. Itin. 2. P. 100)”. Subsequently, he includes a more detailed description and asserts “Habitat prope La Calle”. The only known original material for *B.contortus* is the illustration quoted in Desfontaines (1798: plate 25) and a single sheet in P (MNHN-P P00320328) bearing two specimens mounted on it with a printed label: *Herbier de la FLORE ATANTIQUE donné au Museum par M. DESFONTAINES* and annotated “*Bromus contortus*” handwritten (unknown by whom). There is another label, handwritten by Desfontaines, containing the exact diagnosis and description as it appears in Flora Atlantica (Desfontaines 1798: 95). Maybe Desfontaines is surprised one specimen is very similar to *B.alopecuros*, but not the other one, justifying his doubt in the description where he states “perhaps *B. alopecuros*?” This sheet bears two plants. The one on the left seems to be *Bromuslanceolatus* Roth and the one on the right is a fragment (contracted and erect panicle with subsessile spikelets) of a specimen matching *B.contortus* Desf., that also exemplifies the typical resemblance to *B.alopecuros* Poir. Therefore, the sheet in question does not represent a specimen as defined in the *Code* (Turland et al. 2018); but each of the two plants on the sheet is a specimen in its own right. Only one specimen is original material for *B.contortus* Desf. It is possible that the illustration in Desfontaines (1798: plate 25) was drawn from the plant designated here as lectotype.

Choosing the specimen on the right of the sheet MNHN-P P00320328 (Figure 2) as lectotype, which taxonomically matches *B.alopecuros* Poir., the name becomes a taxonomic or heterotypic synonym (Turland et al. 2018) of it, as Persoon (1805, 1: 95) asserted. Moreover, it is also possible that the heterogeneous material in this sheet is the origin of some misidentifications of *B.lanceolatus* Roth as *B.contortus* Desf.

Later, Poiret (1810: 703) describes *Bromusalopecuroides* “*Bromus* panicula conferta, erecta; spiculis oblongis, pubescentibus, quindecimfloris, subsessilibus; aristis infernè spiraliter contortis”. This description is almost identical to the former of *B.alopecuros*. Its only difference is to include “pubescentibus, quindecimfloris”, the same features Desfontaines (1798: 95) uses to describe *B.contortus*. Poiret continues adding the references to *B.alopecuros* Poir. and the synonym *B.contortus* Desf. Therefore, this name does not need a Lectoype as it is a superfluous and illegitimate renaming (Art. 52. 1) of *Bromuscontortus* Poir. and has the same type we select here for that name.

### Bromus benekenii (Lange) Trimen, J. Bot. 10: 333. (1872)

Taxon classificationPlantaePoalesPoaceae

**Figure 3.**
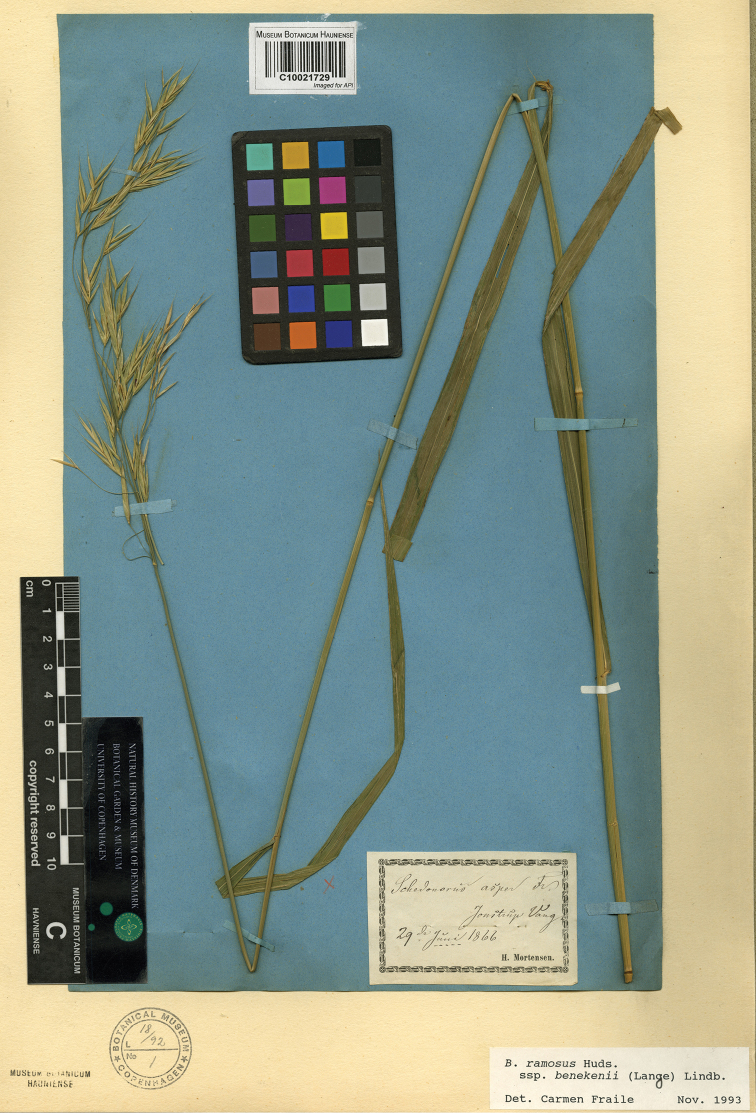
The original specimen of *Schedonorusbenekenii* Lange illustrated in Flora Danica (in table 2826, fig. 2) is the lectotype selected (C10021729). Reproduced with permission of the Natural History Museum of Denmark.

Schedonorus benekenii Lange, Flora Danica 48: 5, t. 2826. (1871). Type Protologue: “In silvia hinc inde. Specimen depictum fig. 1 in insula Lolland legit cl. E. Rostrup, specimen fig. 2 in silva Jonstrup Vang legit cl. H. Mortensen”. Type: [Denmark] Jonstrup Vang. 29^TH^ June 1866, H. Mortensen (lectotype, designated here: C [C10021729 image!]). (Figure 3)

#### Type.

Based on *Schenodorusbenekenii* Lange.

#### Remarks.

The current widely used name *Bromusbenenkenii* is an implicit combination by Trimen (1872: 333) of the name described as *Schedonorusbenekenii* Lange in Flora Danica. In the original publication, Lange (1871: 5) describes a perennial *Bromus* living in forests, with nodding panicle.

An exhaustive search to find the material Lange (1871) cites as models for the illustrations in table 2826 (figs 1, 2), finally had a result. The two syntypes are conserved in C in the Flora Danica subherbarium, that contains specimens drawn in the magnificent work “Flora Danica” (Olof Ryding pers. comm. 2018). Their labels state “insula Lolland legit cl. E. Rostrup” (C10021728)” and “Jonstrup Vang, legit cl. H. Mortensen” (C10021729). Each folder indicates in handwriting that the specimens were drawn for “Flora Danica”.

We choose as lectotype the latter sheet since its spikelets conserve most of its florets. The other sheet is in a more advanced phenological state and conserves, in most of its spikelets, only the glumes. The *Rostrup* specimen still conserves its basal part, with leaves, that is missing in the lectotype.

### Bromus commutatus Schrad., Fl. Germ.: 353 (1806)

Taxon classificationPlantaePoalesPoaceae

Bromus commutatus Schrad., Fl. Germ.: 353 (1806). Type Protologue: “Inter segetes, ad vias, sepes, alibique”. Type: Germany (lectotype designated by Acedo and Llamas 1999: 73): *Bromuscommutatus* Fl. Germ. Göttingen GOET 006096!

#### Remarks.

The lectotype has a handwritten label by Schrader “*B.commutatus / Fl. Germ. / Göttingen*” and a handwritten indication as “typus-material”. Currently, there is another label on this sheet by H.Scholz 1998 marking it as Neotypus, which was published in Scholz (1999: 436). Although both publications fulfil the conditions for a formal typification of a lectotype or a neotype, the Acedo and Llamas (1999: 73) publication appeared in February and is probably the first typification (ICN, Art. 10. 5). There are two important facts to consider. First, according to Kerguélen (1975: 100), there is original material in GOET. We consulted that herbarium and found two sheets that Schrader sent to GFW Meyer, which are indeed original material. Both bear handwritten labels by Schrader. The first sheet label says “*B.commutatus* Fl. Germ. Göttingen” and the second sheet label “*B.commutatus* Fl. Germ. var. spic. paulo brevior. Göttingen”.

### Bromus erectus Huds., Fl. Angl.: 39 (1762)

Taxon classificationPlantaePoalesPoaceae

**Figure 4.**
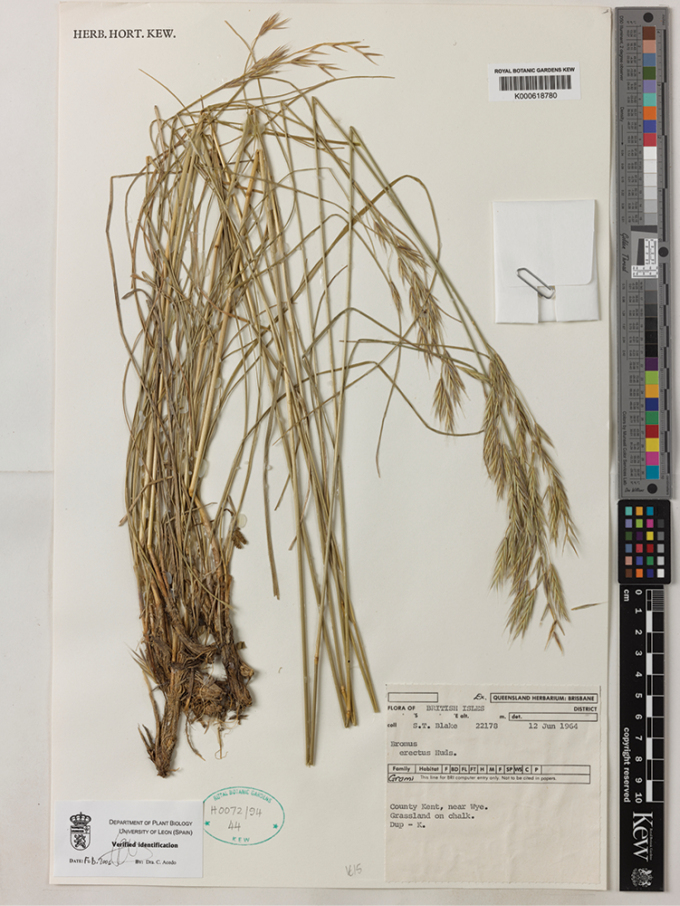
Neotype selected for *Bromuserectus* Huds. Sheet preserved at Herbarium K, barcode K00618780. [Available at http://specimens.kew.org/herbarium/K000618780]. Image used with permission Board of Trustees of the Royal Botanic Gardens, Kew.

Bromus erectus Huds., Fl. Angl.: 39 (1762). Type Protologue: “Habitat in cretaceis circa Rochester, Dartford and Gravesend, in Cantio”. Type: United Kingdom. England: Kent, near Wye, grassland on chalk. 12 Jun. 1964, S.T. Blake 22178 (neotype, designated here: K [K000618780!]; isoneotype: BRI [BRI 252046 image!]). (Figure 4)

#### Remarks.

As a fire in his house destroyed Hudson’s personal herbarium, most specimens were lost and only those borrowed by other botanists are extant. Some specimens which Hudson sent to Linnaeus are preserved in the herbarium of the Linnean Society of London (LINN) but none of them is *Bromuserectus* Huds. There is also one sheet conserved in BM that does not seem to be original material. As Hudson did not give any other reference and, as no original material of *B.erectus* Huds. is available, a neotype must be designated (ICN, Art. 9.13). We searched for material coming from Kent (“Cantium”, England) in K and selected one specimen consistent with the protologue as neotype. It was collected in Kent, near Wye. There is a duplicate of it in BRI, which is an isoneotype.

### Bromus hordeaceus L . subsp. divaricatus (Bonnier & Layens) Kerguélen, Soc. Echange Pl. Vasc. Eur. Bassin Médit., Bull. 18: 27. (1981)

Taxon classificationPlantaePoalesPoaceae

**Figure 5.**
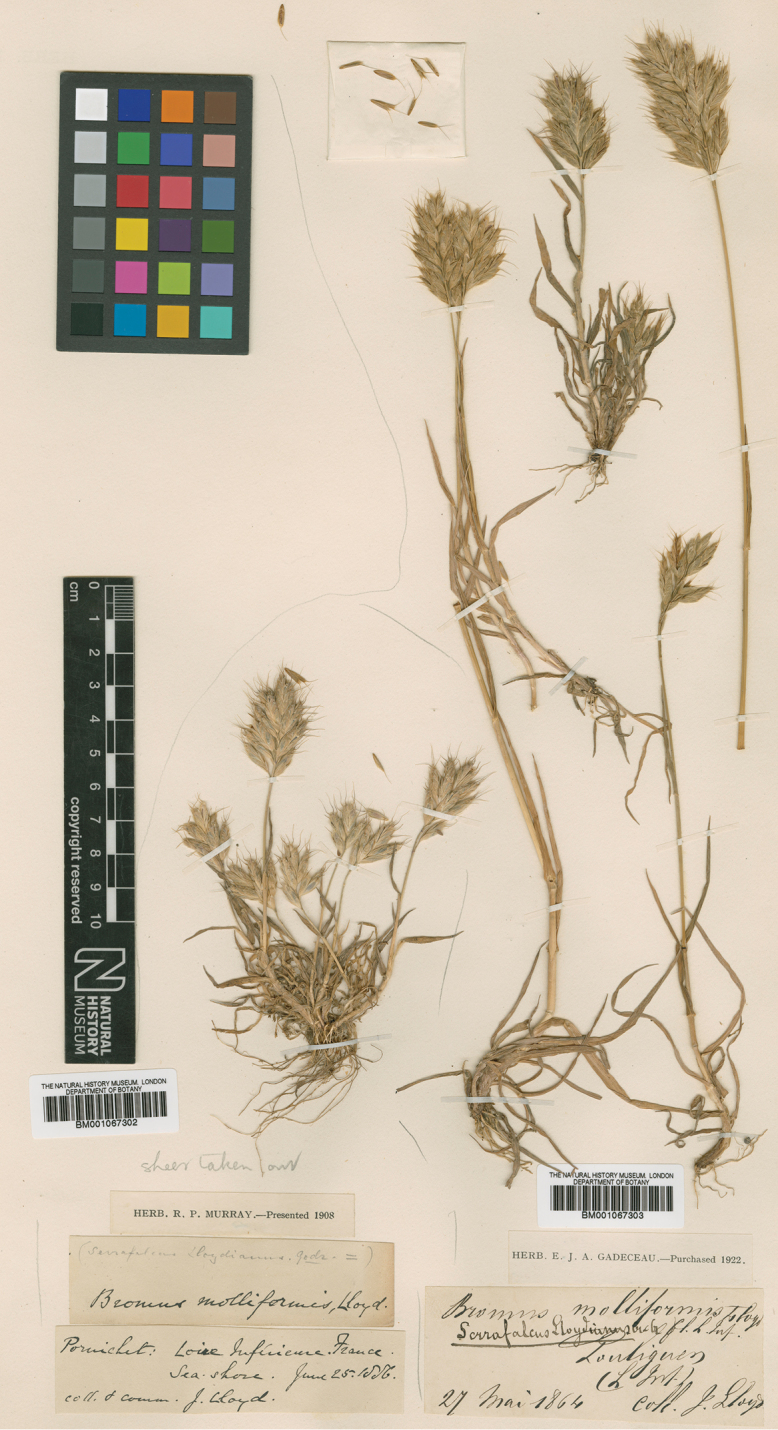
The epitype of BromushordeaceusL.subspdivaricatus (Bonnier & Layens) Kerguélen is the specimen on the left with barcode BM001067302 (collected and identified by Lloyd as *B.molliformis*; preserved at BM (Permanent URL: http://data.nhm.ac.uk/object/9a6e0684-9e9b-4951-ac7e-ba13ad34ed31). Reproduced with permission.

**Figure 6.**
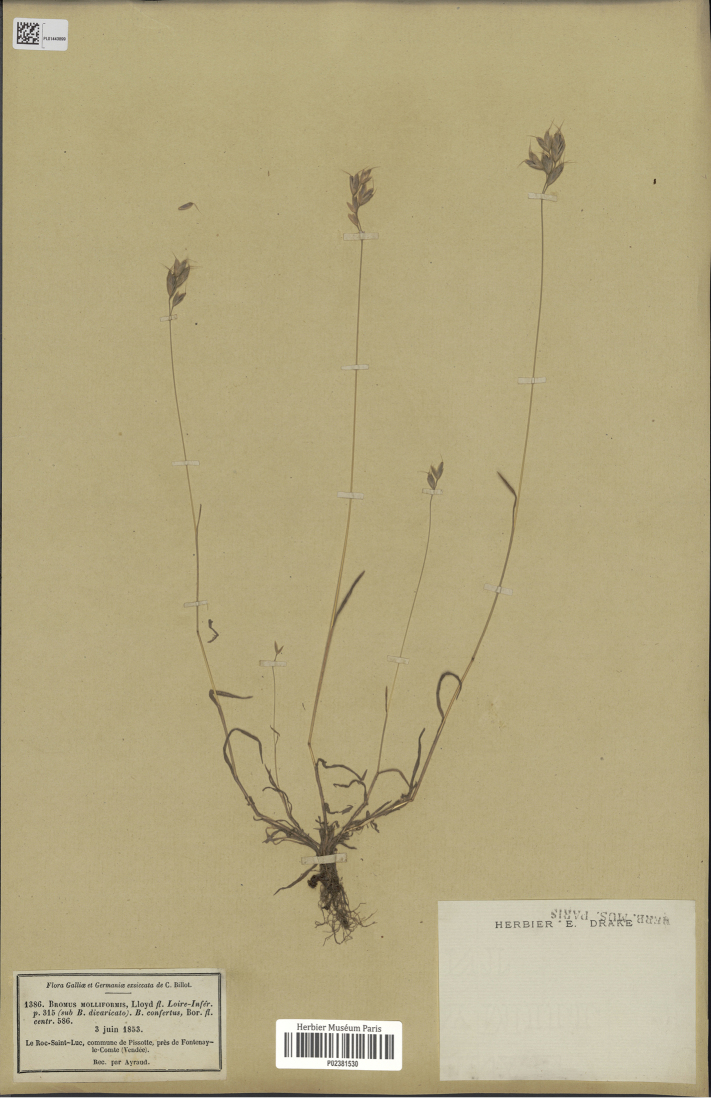
Lectotype for *Bromusmolliformis* J.Lloyd ex Billot at P. It is a specimen preserved at the MNHN Collection Vascular plants. Specimen with barcode P02381530. (Available at http://coldb.mnhn.fr/catalognumber/mnhn/p/p02381530) Reproduced with permission.

Bromus intermedius Guss. subsp. divaricatus Bonnier & Layens, Tabl. Syn. Pl. Vasc. Fl. Fr. 369. (1894), non *B.divaricatus* sensu Rhode ex DC. Type Protologue: [France] “Endroits incultes, sables”. Type: [France], (lectotype, designated here: [figure] in Bonnier and Layens, Tabl. Syn. Pl. Vasc. Fl. Fr. 369. (1894); epitype, designated here: [France]: Pornichet, Loire Inférieure [Loire-Atlantique], sea shore, June 25 1856, Lloyd, BM [BM001067302 image! as *Bromusmolliformis* Lloyd]). (Figure 5)
Bromus molliformis J.Lloyd ex Billot, Fl. Gall. & Germ. Exsicc. (Haguenau) 1: 297–298. (Feb 1854). Type Protologue: “Loire Inférieure” [Loire-Atlantique]. Type: [France]. Le Roc-Saint-Luc, commune de Pissotte, près de Fontenay-le-Comte (Vendée); June 3, 1853, (lectotype, designated here: P [P02381530 image!]; isolectotypes: P [P03354928, P03354936, P03486702, P03364411]). (Figure 6)
Serrafalcus lloydianus Godr. & Gren. in Grenier and Godron, Fl. France 3: 591 (1855). Type Protologue: “Hab. Sables maritimes; Cannes, Hyères, Montpellier, etc.; littoral de l’Océan depuis Bayonne jusqu’à l’embouchure de la Loire”.

#### Type.

Based on BromusintermediusGuss.subsp.divaricatus Bonnier & Layens.

#### Remarks.

*Bromushordeaceus* L. is a very variable species with a complex nomenclatural history at subspecies level. The origin of many problems in the group is the name *B.molliformis* J.Lloyd, Fl. Loire-Inf. (Lloyd 1844: 314–315), which was invalidly published as a provisional name “je proposerais, si c’était une espèce nouvelle, de l’appeler *Br.molliformis*”. The name was later validated as *Bromusmolliformis* J.Lloyd ex Billot, Fl. Gall. & Germ. Exsicc. (Haguenau) 2: Cent. 14. 1854. Billot supplies a brief description for “cette espèce donnée par M.Lloyd dans sa *Flore de la Loire-Inférieure* sous le nom de *B.divaricatitus* Rohde?” Billot (Feb. 1854: 297–298) neglects to mention several details of Lloyd’s description of *B.molliformis* and describes the species as follows:

“Racine fibreuse. Chaume de 2–4 décimètres. Feuilles et graines inférieures mollement poilues. Panicule oblongue, droite, étalée, *resserrée après la floraison*; pédoncules courts, simples. Epillets oblongs, étalés, *velus*; arête égalant la glumelle, d’abord droite, à la fin *tortillée divariquée*, insérée à 1 1/2 millimètre du sommet obtus, échancré”.

Billot (1854) publishes the first validating description for *Bromusmolliformis*. He includes the reference of the features to differentiate the new species from the closely related *B.hordeaceus* L. and from *B.divaricatus* Rhode. He sold his exsiccata to several herbaria. There is not a register of those herbaria. Currently, some of them are included in P where some original specimens with its species number (1386) are preserved. Additionally, there are several names and combinations at several ranks, both validly and invalidly published and various nomenclatural changes. Nevertheless, *B.molliformis* J.Lloyd ex Billot remained untypified and still lacked a stable nomenclature.

*Serrafalcuslloydianus* Godr. & Gren. (1855: 591) is a superfluous name for *B.molliformis* J.Lloyd ex Billot. Grenier and Godron (1855) listed several synonyms, including “*B.divaricatus* Lloyd non Rhode” and *B.molliformis* J.Lloyd ex Billot [sub. *B.molliformis* Lloyd]. They also mention Billot’s exsiccate n. 1586; and include his new species in the complex of species having “arête … tordue sur elle-même et divariquée”. Therefore, this name does not need a Lectoype as it is a superfluous and illegitimate renaming (Art. 52.1) of *Bromusmolliformis* J.Lloyd ex Billot and has the same type we select here for that name.

Bonnier and Layens (1894: 369) describe BromusintermediusGuss.subsp.divaricatus Bonnier & Layens, based on *B.divaricatus* [sensu] Lloyd, non Rhode, with the characteristics of Lloyd’s plant. Kerguélen (1981: 27) combines it to Bromushordeaceussubspdivaricatus (Bonnier & Layens) Kerguélen. Bonnier and Layens type material is unknown (Stafleu and Cowan 1976). Kerguélen (1975: 104) mentions Lloyd’s type material is in herbarium NTM, pointing out two localities “Pornic, Saint Brevin” indicated by Lloyd. Nevertheless, no original material is preserved in NTM (Mary Laury Guerin, Com. pers. 2018).

BromushordeaceusL.var.molliformis (J.Lloyd ex Billot) Halácsy (1904: 396). This is another combination of *B.molliformis* J.Lloyd and Halácsy pointed out its main traits “Panicula magis conferta, ramis brevissime, spiculis densius et longius velutinu-pilosis, aristis demum extrorso subcurvatis”.

BromushordeaceusL.subsp.molliformis (J.Lloyd) Maire & Weiller (1955: 255). This post-1953 combination, without direct citation to the basionym, nor to a potentially validating Latin description, but only to “*Bromusmolliformis* Lloyd, Fl. Loire-Inf. 315 (1844)”, is another combination not validly published.

As Bromusintermediussubsp.divaricatus Bonnier & Layens is an untypified name, we choose, as lectotype, the figure in Bonnier and Layens (1894: 369) that is definitely original material. However, as is common with figures, it is difficult to observe some diagnostic characteristics. Thus, we select, as epitype, a specimen collected by Lloyd, conserved in BM, consistent with the protologue. It is a sheet bearing two collections. We choose the specimen on the left from Pornichet (barcode BM001067302).

### Bromus lepidus Holmb., Bot. Not. 1924: 326 (1924)

Taxon classificationPlantaePoalesPoaceae

**Figure 7.**
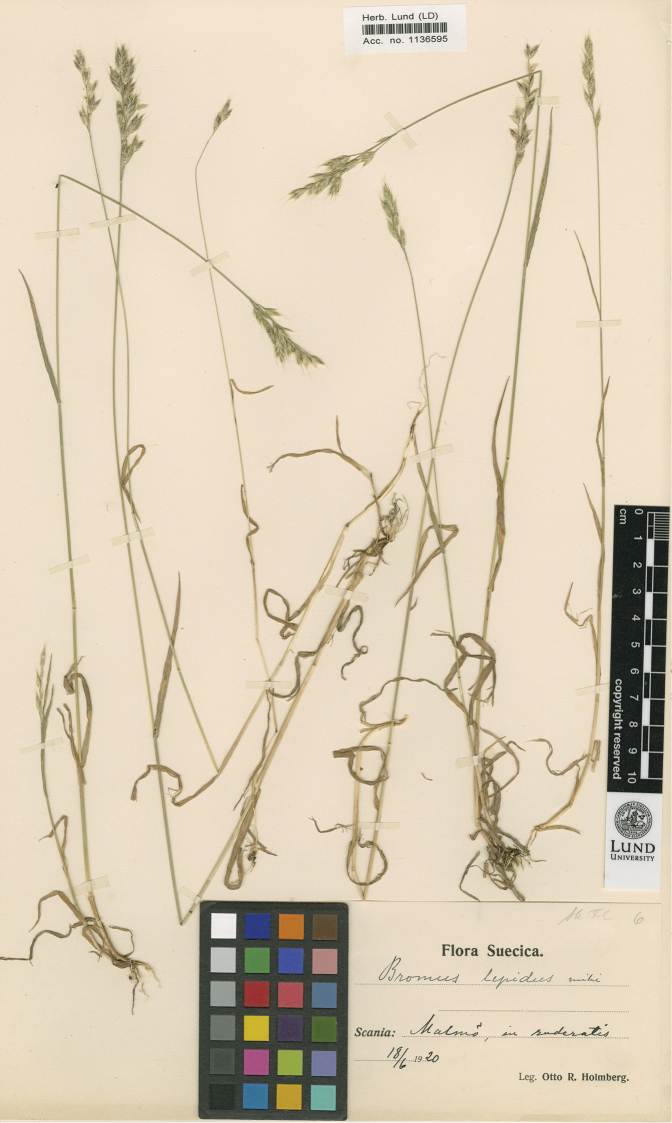
Lectotype of *Bromuslepidus* Holmberg, preserved at the Herbarium of the biological museum of the Lund University (LD1136295), reproduced with permission.

**Figure 8.**
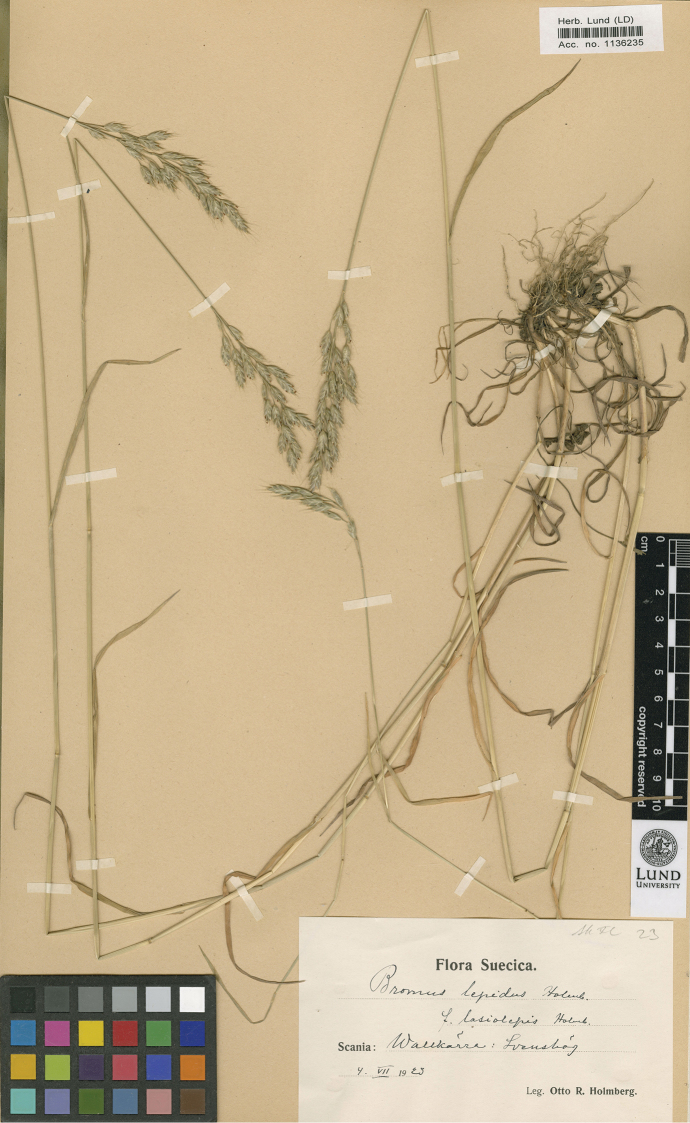
Lectotype of Bromuslepidusf.lasiolepis Holmberg, preserved at the Herbarium of the Biological Museum of Lund University (LD 1136235). Reproduced with permission.

Bromus lepidus Holmb., Bot. Not. 1924: 326 (1924).Type Protologue: “Nach ROUY kommt sie in Frankreich hier und da vor,aber ziemlich selten; Krösche hat sie nur von einem Ståndort in Norddeutschland. In Schweden kommt sie besonders in Schonen vor; ich habe sie mehrmals eingesammelt, wie in der Gegend von Lund und Malmö an mehreren Orten; Svenshög in Wallkärra; Skartofta in Öved; Gudmundtorp. Außerdem sah ich Exemplare aus Blommeröd in Öved (leg. P. Boren 1903), Skelderviken (leg. Sten Selander), Kalmar (leg. N. Blomgren), Borås (leg. C. Sandberg), Fyen, Stenlose (leg. G. Samuelsson). Kommt in Klee- und Grasfeldern, auf Rainen, Wegrändern etc. vor, oft mit *B.mollis* und *B.commutatus* zusammen; bei Malmö auch als Ruderat“. Type: SWEDEN. Scania: Malmö, in ruderatis, 18-06-1920, Otto R.Holmberg, (lectotype, designated here: LD [LD1136595 image!]; isolectotype: K [K000913599!]). (Figure 7)
Bromus lepidus Holmb. f. lasiolepis Holmb., Bot. Not. 1924: 326 (1924). Type Protologue: not indicated. Type: Sweden. Scania: Vallkörra. Svenenshög. 4-07-1923. Otto R.Holmberg, (lectotype, designated here: LD [LD 1136235 image!]; isolectotype: K [K000913598!]). (Figure 8)

#### Remarks.

This taxon has a long history full of nomenclatural problems. Duval-Jouve (1865: 208), who only lists, without naming them, the variations in spikelet size and hairiness of *Bromus* species, e.g. *Bromusmollis* “microstachys glabre” and *Bromusmollis* “microstachys pubescent”. This is the first mention of this taxon. Later Rouy (1913: 236) proposed a named variety under the genus *Serrafalcus* Parl.: *S.mollis* ß *microstachys* Rouy, giving “*Bromusmicrostachys*Duval-Jouve” (1865: 207) as a synonym and adding a diagnosis. Therefore, there is no doubt this is the same plant cited by Duval-Jouve (l. c.). Afterwards, Krösche (1924: 329) described *B.gracilis* Krösche, which unfortunately is a posterior homonym as Weigel (1772: 15) previously proposed this name for a different plant.

Finally, Holmberg (1924: 326) solves this unfortunate situation and names simultaneously the new taxa *Bromuslepidus* Holmb. and B.lepidusf.lasiolepis Holmb. The herbaria having Holmberg material are K, LD and S. There are 13 sheets in LD collected before 1924, five in S and three in K. It is reasonably certain that Holmberg studied all those plants before the description of the species and form and that all of them are original material. Therefore, we decided to limit our choices to sheets with the annotations “*B.lepidus* mihi” and “B.lepidusf.lasiolepis Holmb.”, as this annotation indicates that Holmberg is interpreting them as the new taxa he is going to describe.

### Bromus ramosus Huds., Fl. Angl.: 40 (1762)

Taxon classificationPlantaePoalesPoaceae

**Figure 9.**
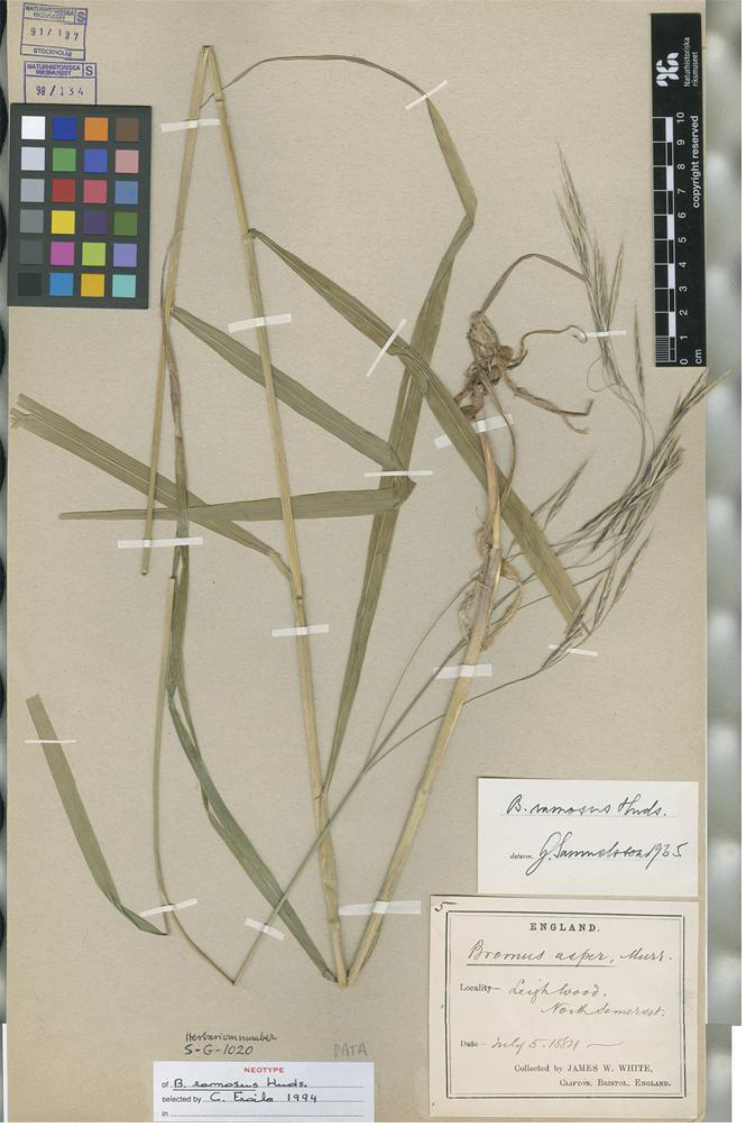
Neotype selected for *Bromusramosus* Huds. preserved at the general collection of the Swedish Museum of Natural History (S) (S-G-1020!). Reproduced with permission.

Bromus ramosus Huds., Fl. Angl.: 40 (1762).Type Protologue: “Habitat in sylvis et sepibus frequens”. Type: United Kingdom. England: Leighwood, North Somerset. 5 July 1884, White, J.W #s.n., (neotype, designated here: S [S-G-1020!]). (Figure 9)

#### Remarks.

Hudson (1762: 40) proposed the name *Bromusramosus* for the wood Brome-grass from England. The protologue is a short diagnosis “*BROMUS* panicula ramosa nutante scabra, spiculis linearibus decemfloris arista longioribus, foliis scabris” followed by three polynomials as “synonyms”, but without indication of the geographical area in which the new species lives, except an indication to “Anglia”. As pointed out before, the fire destroyed Hudson’s house as well as his personal herbarium. We were not able to find any sheet that could be “original material”. Accordingly, we select a neotype of *Bromusramosus* Huds. An annotation label indicates that Carmen Fraile verified and chose it as a neotype in 1994, but this designation was never published (ICN, Art. 7. 10). As this sheet agrees with the protologue, we accept her choice and make it effective here.

### Bromus subg. Stenobromus (Griseb.) Hack. in Engler & Prantl, Nat. Pflanzenfam. 2(2): 75. 1887

Taxon classificationPlantaePoalesPoaceae

≡ Bromussect.Stenobromus Griseb., Spic. Fl. Rumel. 2: 448 1844 [1846].

#### Type.

(designated here): *Bromussterilis* L., Sp. Pl.: 77 (1753).

#### Remarks.

Grisebach (1844: 448) described Bromussect.Stenobromus including *B.maximus* Desf., *B.madritensis* L., *B.tectorum* L. and *B.sterilis* L. Hackel (1887: 75) described B.subg.Stenobromus including only two species: *B.tectorum* L. and *B.sterilis* L and gave *Anisantha* K. Koch as a synonym. Afterwards, some other species were included. Until now, B.subg.Stenobromus has been used in some works (Stebbins 1981, Acedo and Llamas 1999) because there is no correct name available for this taxon at subgenus level. As a consequence, B.subg.Stenobromus is treated as a new combination at new rank because a potential basionym exists, B.sect.Stenobromus, applying to the same taxon ICN Art. 41.4.

This subgenus is variable enough to have two sections:

### 1. Bromus sect. Genea Dumort., Observ. Gramin. Belg. 116 (1823) [1824]

Taxon classificationPlantaePoalesPoaceae

#### Type.

(designated by Tournay 1961: 294): *Bromussterilis* L. in Sp. Pl. 77 (1753).

#### Included species.

Dumortier (1824) describes the section including *Bromussterilis* L., *B.tectorum* L., B. *diandrus* Roth, *B.rigidus* Roth. and *B.rigens* L., a synonym for *B.scoparius* L., now belonging to B.sect.Triniusa (Steud.) Nevski.

#### Distribution.

Europe, temperate Asia and North Africa.

### 2. Bromus sect. Penicillus Llamas & Acedo, sect. nova.

Taxon classificationPlantaePoalesPoaceae

urn:lsid:ipni.org:names:77196853-1

#### Diagnosis.

Panicle dense, compact, with short branches. The erect spikelets seem to be sessile. Awns more or less divaricate at maturity.

#### Type.

*Bromusrubens* L. in Cent. Pl. I: 1 (1755).

#### Included species.

*B.rubens* L., *B.fasciculatus* C.Presl and *B.matritensis* L.

#### Distribution.

Southern Europe, temperate Asia and North Africa. Etymology: Named from the Latin “penicillus”, meaning brush, referring to the morphology of the inflorescence, especially in *B.rubens*, the name of which provides the type.

## Supplementary Material

XML Treatment for Bromus alopecuros Poir., Voy. Barbarie. 2: 100–101 (1789)

XML Treatment for Bromus benekenii (Lange) Trimen, J. Bot. 10: 333. (1872)

XML Treatment for Bromus commutatus Schrad., Fl. Germ.: 353 (1806)

XML Treatment for Bromus erectus Huds., Fl. Angl.: 39 (1762)

XML Treatment for Bromus hordeaceus L . subsp. divaricatus (Bonnier & Layens) Kerguélen, Soc. Echange Pl. Vasc. Eur. Bassin Médit., Bull. 18: 27. (1981)

XML Treatment for Bromus lepidus Holmb., Bot. Not. 1924: 326 (1924)

XML Treatment for Bromus ramosus Huds., Fl. Angl.: 40 (1762)

XML Treatment for Bromus subg. Stenobromus (Griseb.) Hack. in Engler & Prantl, Nat. Pflanzenfam. 2(2): 75. 1887

XML Treatment for Bromus sect. Genea Dumort., Observ. Gramin. Belg. 116 (1823) [1824]

XML Treatment for Bromus sect. Penicillus Llamas & Acedo, sect. nova.
